# Adherence to Isoniazid Preventive Therapy among Under-Five Children in Contact with Adult Bacteriologically Confirmed Pulmonary Tuberculosis Patients: A Mixed-Method Study

**DOI:** 10.1155/2020/8834806

**Published:** 2020-09-29

**Authors:** Getachew Seid, Tsegaye Tsedalu, Marta Ayele

**Affiliations:** ^1^Ethiopian Public Health Institute, Addis Ababa, Ethiopia; ^2^Wollo University, Dessie, Ethiopia; ^3^Addis Ababa Health Bureau, Addis Ababa, Ethiopia

## Abstract

**Background:**

The World Health Organization recommends that all children below the age of five who have household contact with an infectious tuberculosis case should receive isoniazid preventive treatment for at least six months after the active tuberculosis disease has been ruled out. This research aims to determine the adherence of children, eligible for isoniazid preventive treatment, to the treatment who had contact with pulmonary tuberculosis patients.

**Methods:**

A mixed study design was used to prospectively assess the adherence to IPT among children under the age of 5 in contact with pulmonary TB patients through the quantitative study design and barriers of adherence in view of health care professionals and the family of children through a descriptive qualitative study. The study was conducted from July 2019 to December 2019 in Addis Ababa. Data were collected by a structured datasheet from the selected health center registration book. Data were entered into Epi Data software and analyzed by using SPSS version 20. Descriptive statistical methods were used to summarize the sociodemographic characteristics of the study participants.

**Result:**

The ratio of the total number of pulmonary tuberculosis index cases recruited into the study to the number of child contacts aged less than 5 years was 1 : 1.32. The total isoniazid preventive treatment uptake in this study was 75.2%; one-fifth (21.3%) of the children who started IPT did not complete the full course of six-month isoniazid preventive treatment. Except for HIV not to be tested (*P* < 0.001), there was no significant association of the listed risk factors in default to complete the full six months of preventive treatment.

**Conclusion:**

Enrolment of eligible children for isoniazid preventive treatment in the urban city Addis Ababa was still below the target of the World Health Organization End tuberculosis strategy by 2030. The treatment adherence rate also needs a great deal of effort to achieve the strategy. Child default after the first visit indicates a lack of understanding about the benefit and safety of preventive therapy in young children among families of TB patients, and awareness-creating efforts by health extension workers will help to improve the outcomes.

## 1. Background

Tuberculosis is highly infectious; a person with active TB may be able to infect up to 15 people a year by being in close contact. Tuberculosis, one of the top ten causes of death, is a significant contributor to the international and the national burden of disease [1].

According to figures from the World Health Organization (WHO) in 2018, 10% of the 9 million cases of tuberculosis (TB) occurred in children, resulting in 210,000 TB-related deaths, including 170,000 in HIV-negative children. Children exposed to index cases of TB, particularly sputum smear-positive (PTB) ones, are at risk of infection and are at high risk of developing the disease when infected with tubercle bacilli, especially infants and young children (<5 years of age) [2].

Ethiopia is also one of the 22 high-burden countries for TB, and childhood TB accounts for 10% of the total TB burden with cases reporting among children under the age of 15 estimated at 11,361 [3].

The WHO recommends in its global guidance, for post 2015, the need for preventive treatment of high-risk people as one strategy for prevention, care, and control of TB [4]. The WHO also recommends that all children under the age of five who have household contact with an infectious TB case should be given IPT for at least six months after the active TB disease has been ruled out [5].

To identify children with TB disease and enable them to treatment early, the WHO recommends screening the household contacts of an infected source case, which allows providing isoniazid preventive treatment (IPT) for household contacts who do not have the disease [6]. It has been shown earlier that, as a preventive measure, regular isoniazid (INH) administration reduces the likelihood of progression of latent tuberculosis infection (LTBI) to active TB disease in young children for at least six months [7]. Isoniazid chemoprophylaxis therapy can reduce the risk of contracting tuberculosis by 59% in children aged 15 or younger [8]. IPT is effective in reducing the incidence of tuberculosis and is included in the National TB Program (NTP) recommendations of several resource-limited countries [9].

Even though IPT is a global recommendation and many countries endorsed it in their national strategy, its initiation and completion rates are suboptimal. Some of the factors which affect IPT prophylaxis service were the level of awareness among health care providers, interruption of INH supply, co-infection with HIV, lack of recording tools for IPT, and distance from health facilities.

While the IPT initiation and completion rates recorded in program implementation settings ranged from 21% to 58% and 13%, respectively, the WHO still recommends the use of IPT [10]. Nevertheless, IPT is not routinely prescribed to at-risk children and is often unattended and characterized by insufficient uptake and adherence when provided. IPT's efficacy relies on 80% or greater adherence to medication [8].

Ethiopia has also established a national strategy for the prevention and control of TB in infants, emphasizing the implementation of screening and the provision of IPT for children under 5 years of age as one intervention to prevent TB in infancy. According to Ethiopian guidelines, after excluding the involvement of active TB disease in children under the age of five years who have a history of contact with a patient with bacteriologically confirmed pulmonary TB, IPT should be provided for six months [11].

The prevention of TB among contacts of TB source cases depends on their willingness to accept and adhere to a chemoprophylaxis regimen. According to the Ethiopian ministry of health 2018 report, in Ethiopia, from the total screened under-five children, only 65% of them started chemoprophylaxis for TB [12].

Most studies conducted on IPT focus on the setting of TB/HIV co-infected populations and children in a rural area. We present the IPT uptake and completion rate implementation experience under routine program intervention in the urban setting of Addis Ababa, Ethiopia. Hence, the objective of the study was to assess the adherence to IPT among children under the age of 5 in contact with pulmonary TB patients who were eligible for prophylaxis.

## 2. Methods

### 2.1. Study Settings

The study was conducted in Addis Ababa, the capital of Ethiopia, inhabited by about 4 million people, the majority being of urban origin [13]. The city has more than 141 public health centers (PHCs) in its 10 subcity offering tuberculosis diagnosis and treatment for patients with tuberculosis [14]. All of these public health centers give IPT for children under 5 years of age in contact with pulmonary positive tuberculosis patients.

### 2.2. Study Design and Participants

The mixed study design was used to prospectively to assess adherence to IPT among children under the age of 5 in contact of pulmonary TB patients through the quantitative study design and barriers of adherence in view of health care professionals and family of children through a descriptive qualitative study.

The target population was children under 5 years of age who had contact with pulmonary positive tuberculosis patients, whereas the study population was children under 5 years of age who were eligible for IPT treatment. For the qualitative study, all parents and the health care professionals who gave treatment for TB patients and eligible children were interviewed.

A bacteriologically confirmed pulmonary TB patient is defined as a patient with either at list one smear positive or positive in gene Xpert MTB RIF assay. A household contact for this study was defined as a child aged below 5 years who lives or has lived within the household of bacteriologically confirmed pulmonary TB case.

A child below the age of 5 years who was in contact with a bacteriologically confirmed pulmonary TB patient within the same household within 3 months before the patient was diagnosed was eligible for the study by ruling out the disease. After the parents signed the written informed consent, the children were enrolled. Ineligible child contacts included those that were not living in the same household with the index cases before the diagnosis and child contacts older than 5 years.

Within the study period, 184 index cases of pulmonary-positive TB patients were diagnosed and treated at the selected site. Of the 184 index cases, only 173 whose child contacts started IPT have been recruited for this study that was conducted between July 2019 to December 2019; of these, 94 (54.3%) had at least one child contact who was under 5 years old at the time of the study. The 94 index cases had 129 child contacts.

### 2.3. Study Variables

Sociodemographic and other variables of under 5 children like HIV status, relation to the index patient, BCG vaccination child and index, pulmonary positive TB patient's treatment history, number of child, and other variables were collected by using structured questionnaires.

### 2.4. Definition of Variables

Complete adherence in this study is defined as the collection of six of the child's monthly prescriptions. Incomplete adherence means that the child had received/collected less than six of his/her monthly prescriptions.

### 2.5. Sampling Procedure

The study was conducted at six public health centers (PHCS) providing TB diagnostic and treatment services in Addis Ababa from December 15, 2018, to March 15, 2019. The criteria used to select six PHCs from the total PHCs providing TB diagnostic and treatment as well as IPT services in the area were a record of at least 8 bacteriologically confirmed pulmonary tuberculosis patients in their registration in the last quarter before the study began. All consecutive pulmonary positive TB patients who meet the eligibility criteria were included in the study.

### 2.6. Data Collection and Quality Assurance

The questionnaire was developed, pretested during a pilot study in the selected sites, and modified for later use in data collection. This questionnaire was used to record each time the child came to collect a month's supply of IPT. During follow-up, the trained data collectors and TB clinic nurses monitored all eligible child contacts for symptoms suggestive of TB.

Two health professionals experienced in qualitative methodology were recruited to conduct interviews in the local language using interview guides designed for this specific study. They interviewers investigated factors of IPT inadherence, expectations, and suggestions. For the qualitative part, the trained data collectors carried out in-depth interviews and audio-recorded with 19 parents of child contacts and nineteen health-care providers working in the TB service. Purposive sampling technique was used to select parents and health care workers from the selected public health centers to represent child contacts with IPT adherence. The interview was transcribed by the two health professionals, and then the principal investigator translated the transcribed data.

To ensure the quality of the collected data, one-day training was given to data collectors on the objective, methods, tool, and ethics of the study. The overall activity was monitored by the principal investigator and co-investigators daily.

### 2.7. Data Management and Analysis

EpiData software was used to double enter, verify, and analyze data. Descriptive and multivariate statistics were used and categorical variables were described using frequency tables and proportionate methods. The relation with key analytical outputs was determined using odds ratio and confidence intervals (CIs) of 95 percent. Qualitative data were analyzed using thematic analysis.

### 2.8. Ethical Consideration

Ethics approval was obtained from the Addis Ababa Ethical Review Board. Parents or caregivers of children were interviewed after explaining the objectives of the study, and obtaining written informed consent in local language (Amharic).

## 3. Result

**Figure 1:**
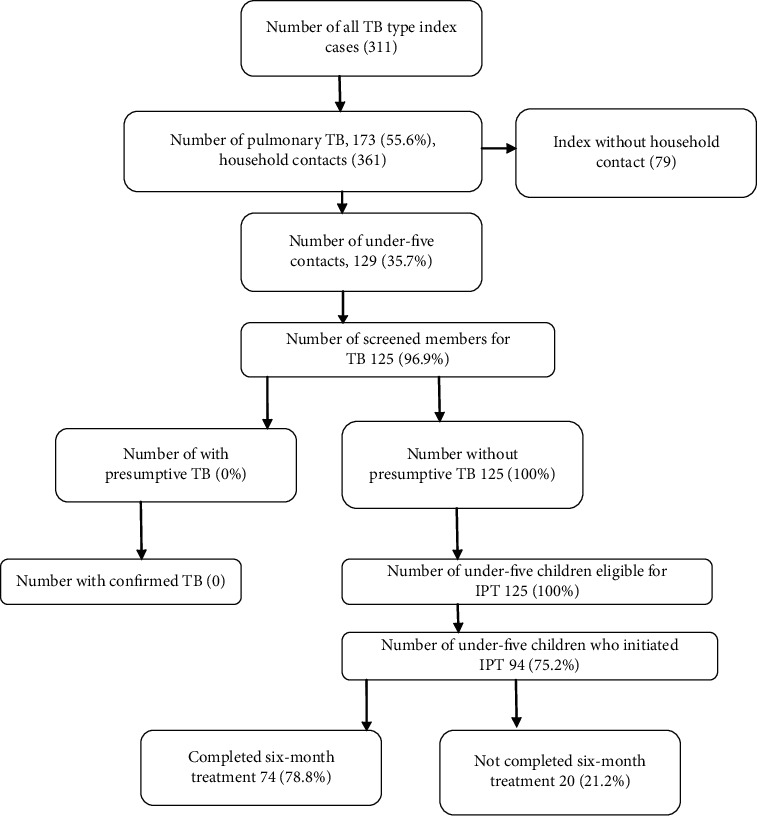
Under-five child contact screening in Addis Ababa health facility, 2019.

At the selected health center registration, a total of 311 TB patients were registered in the study period Of these, 184 were pulmonary-positive TB patients. These included children and adults with pulmonary and extrapulmonary disease. One-hundred seventy-three pulmonary index patients (PTBs) who met the inclusion criteria were included in the study and reviewed; only 94 had at least one child below 5 years in their household. From these index cases, a total of 129 under-five child contacts were identified. There was no presumptive TB case from the screened children. The ratio of the total number of pulmonary TB index cases recruited into the study to a number of child contacts aged less than 5 years was 1 : 1.32. The total number of children on IPT regime in this study was 75.2% (Figure 1).

Almost above half of the pulmonary TB patients were aged between 18 and 30 years (57.4%), were female (58.5%), and had household contact less than three (51.0%). The majority were new TB cases (87.2%), linked by themselves to the health facility (81.9%), and HIV negative (85.1%). The mean age of the pulmonary TB patients that had child contact less than the age of 5 was 34.0 (±13.8) (Table 1).

Around one-fifth (21.3%) of the children who started IPT did not complete the full six months of preventive treatment. The majority of IPT-started children were BCG vaccinated (86.2%), index cases (87.2%), and not HIV tested (84.0%). The mean age of under-five child contact was 3.11 (±1.16) (Table 2).

From the qualitative data by thematic analysis, the reasons given by health workers for not completing prophylaxis who were on IPT were lack of awareness of the family by saying the following:

“*Why my healthy baby takes a drug. My child psychology may be damaged when he takes a drug for long time so do not want to give him/her for a long time.*”

The other one is that the parents said that

‘*My child is too harsh to come to clinics and he/she is unwilling to take the drug for a long time and the drug may have side effects on the future on my child.”*

By multivariate analysis, there was no significant association of not completing the INH prophylaxis with the listed risk factors except for HIV not tested group (*P* < 0.001) (Table 3).

**Figure 2:**
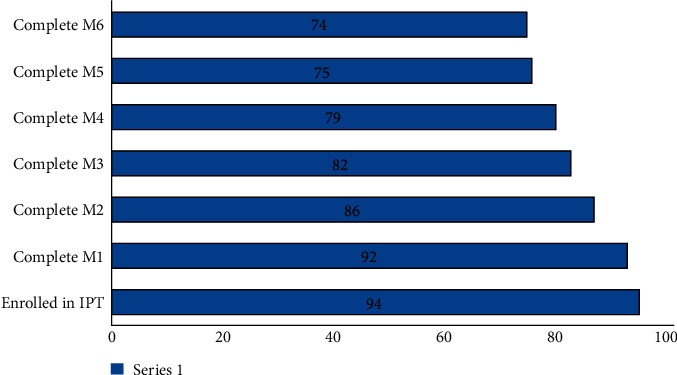
The number of retained children under the age of 5 at each month of INH prophylaxis treatment in Addis Ababa. ^*∗*^Complete M, those children who complete the monthly treatment.

Among the children who interrupted IPT treatment (*n* = 20) were 2 children in the first month, 6 children in the second month, 4 children in the third month, 3 children in the third month, and 1 in the sixth month. More than three-fourth of (78.7%) of the children who initiated IPT completed the six-month prophylaxis treatment (Figure 2).

As health workers on the IPT treatment perception, the reason why children mostly interrupt the prophylaxis treatment was that the family said “no change seen in my child” (78.9%), index patient transfer out (15.8%) and side effect of the drug on the child (5.3%).

## 4. Discussion

Globally, following the adoption of the End TB Strategy by the international community, WHO recommends screening of household contacts of bacteriologically confirmed index cases of active TB, additionally to provide preventive treatment for contacts without TB disease that are susceptible to developing disease following recent infection below the age of 5 years [5]. So far, the least implemented action is TB preventive treatment [4].

Our study found a PTB index case to child contact ratio of 1 : 1.32. This ratio was higher than that recommended by the WHO TB treatment guidelines [15]; for every TB index case diagnosed, at least one child contact was aged less than 5 years (1 : 1). The reason behind was that Ethiopia is one of the developing countries with a high population of people aged less than 14 [16].

The IPT enrolment established in this study was found to be higher than that reported in Malawi, Timor-Leste, South Africa, South India, and Ethiopia: 6% [17], 18.7% [18], 26.8% [19], 33% [20], and 64.3% [21], respectively. This is due to the time difference that the studies were conducted; now a days, globalization has a great impact on information sharing which increase the knowledge of family on different things like the purpose of IPT by electronic and social media.

In contrast, recent studies conducted in the Gambia [22] and Benin [23] have reported 89% and 99% of IPT uptake, which were higher than this study's findings, respectively. When we compared with the Ethiopian finding [21], our IPT enrolment result was higher; the reason was that our study was performed in the capital urban city of Ethiopia, which gives easy access to a health facility to parents when compared with the rural part of Ethiopia. The other reason was that index cases in urban had more knowledge about health-related issues which was supported by the accessibility of health information through the different systems like fliers and posters.

Studies indicated that IPT initiation rates in Africa range from 18% to 68% [21]. The 2006 recommendations of the Wolfheze conferences for contact investigation state that at least 85% of contacts with LTBI should be put on treatment with a minimum of three-fourths of them completing the treatment [1].

In our study, only three-fourth of eligible children successfully completed the recommended IPT dose. The IPT adherence rate in this study was much higher than that reported from southern India and that reported from another study in southern Ethiopia [24]. In Pakistan, of 184 under-five children enrolled in IPT, 60 (32.6%) completed six months of IPT [25]. But, in the South African report [18], only 15% achieved four months of therapy. But, this finding was a little smaller than that from Ethiopia (80%) [21]. Even though it is the best achievement, it still needs a great effort to score the 100% completion rate.

Even though it was still lower than the End TB Strategy of WHO, compared with the rural part of Ethiopia, we showed that there was a high completion rate of preventive treatment for six months in children. Urban health extension workers had their contribution to preventive treatment as they frequently interact with the community, house to house. Reducing factors that hinder the further increase of PT completion rates is crucial to decrease the burden of disease.

The majority of children drop out in a time period between initiation and the first follow-up visit. The higher dropout rate in the first month could be explained by the fact that most of the patients understand and apply the advice of health workers to initiate the treatment; through time, due to many reasons like negligence, cultural intervention, and other impacts, prevention treatment that was started was discontinued. Tools to enhance completions should include community-based teaching and social mobilization.

There was an interruption of preventive treatment each month, even in the sixth month. This might be due to family's lack of awareness on IPT, socioeconomic impact, and unwillingness of children to take a drug. Health workers in TB clinics could give much time to awareness creating on IPT purposes both to the child and the family. These data suggest that the National TB Program strengthens and expands contact management with a community-based approach and incentives.

Incomplete adherence to IPT in this study was not associated with any individual characteristics of index cases as in other studies [16, 17]. However, the small sample size is a limitation of this study that may have limited the ability of researchers to calculate association.

In general, 74 individuals completed their therapy. Thus, assuming a mean protective eﬀect of 75% (range 60–90%) [4] and a 5–10% life-time risk of LTBI reactivation [25], the prescribed PT might have prevented 4–8 new TB cases.

The study has some limitations. First, the sample size was small which affected our statistical analysis on IPT enrolment and completion rate. Second, the development of TB among those who completed IPT was not assessed due to a shortage of information.

## 5. Conclusion

Enrolment of isoniazid preventive treatment for eligible children, in the urban city Addis Ababa, was still below the target of the WHO End TB strategy by 2030. The treatment adherence rate also needs a great deal of effort to achieve the strategy. from preventive treatment after the first visit indicates a lack of understanding about the benefit and safety of preventive therapy in young children among families of TB patients, and awareness-creating efforts by health extension workers will help to sustain PT adherence.

## Figures and Tables

**Table 1:** Characteristics of index cases of the child contacts by uptake of IPT, 2019.

Index characteristics	Index cases (*N* = 94) (%)	Index case whose children were administered IPT (*N* = 73) (%)	Index case whose children were not administered IPT (*N* = 21) (%)
Age group			
18–30	54 (57.5)	44 (60.3)	10 (47.6)
31–45	24 (25.6)	17 (23.3)	7 (33.3)
>45	15 (15.9)	12 (16.4)	4 (19.1)
Sex			
Male	39 (41.5)	29 (39.7)	10 (47.6)
Female	55 (58.5)	44 (60.3)	11 (52.4)
Category			
New	82 (87.2)	64 (87.7)	18 (85.6)
Relapse	12 (12.8)	9 (12.3)	3 (14.4)
Link			
HEW	2 (2.1)	1 (1.4)	1 (4.8)
PPM	15 (16.0)	10 (13.7)	5 (23.8)
PHF	77 (81.9)	62 (84.9)	15 (71.4)
HIV result			
Positive	14 (14.9)	11 (15.1)	3 (14.3)
Negative	80 (85.1)	62 (84.9)	18 (85.7)
Household member			
<3	48 (51.1)	38 (52.1)	10 (47.6)
>3	46 (48.9)	35 (47.9)	11 (52.4)

^*∗*^Isoniazid prophylaxis treatment. PHF, directly to the public health facility by themselves. PPM, linked to TB clinic through public private members (NGOS and private clinics). HEW, linked to TB clinic through health extension worker.

**Table 2:** Characteristics of child contacts after completion of six-month INH chemotherapy, 2019.

Child/index case characteristics	Total children on IPT *N* = 94 (%)	Children who completed IPT 74 (78.7%)	Children who did not complete IPT *N* = 20 (21.3%)
Age group			
<2	33 (35.1)	25 (33.8)	8 (40.0)
2–3	25 (26.6)	21 (28.4)	4 (20.0)
3–5	36 (38.3)	28 (37.8)	8 (40.0)
Sex			
Male	39 (41.4)	41 (55.4)	7 (35.0)
Female	55 (58.6)	33 (44.6)	13 (65.0)
BCG scar			
Yes	81 (86.2)	64 (86.5)	17 (85.0)
No	13 (13.8)	10 (13.5)	3 (15.0)
Relation with index			
Child	82 (87.2)	64 (86.5)	18 (90.0)
Others	12 (12.8)	10 (13.5)	2 (10.0)
HIV tested			
Yes	15 (16.0)	10 (13.5)	5 (25.0)
No	79 (84.0)	64 (86.5)	15 (75.0)

^*∗*^Isoniazid prophylaxis treatment.

**Table 3:** Risk factors for noncompletion of INH prophylaxis in Addis Ababa city, 2019.

Child characteristics	AOR (95% CI)	*P* value
Age group		
<3	8.517 (0.492–147.427)	0.141
Sex		
Female	1.198 (0.267–5.36)	0.814
BCG scar		
Yes	0.968 (0.142–6.577)	0.973
HIV tested		
No	—	**<0.001**
Relation with index		
Child	1.544 (0.118–20.20)	0.741
Index characteristic		
Sex		
Female	0.788 (0.167–3.720)	0.765
Category		
New	493 (0.055–4.413)	0.527
Link		
PHF	0.345 (0.050–2.370)	0.279
No. of under-five children		
1	0.204 (0.005–9.054)	0.411

## Data Availability

The datasets used and/or analyzed during the current study are available from the corresponding author on reasonable request.

## Abbreviations

BCG: Bacillus calmette–Guerin
CIs: Confidence intervals
HIV: Human immunodeficiency virus
INH: Isoniazid
LTBI: Latent tuberculosis infection
NTP: National TB Program
PTB: Pulmonary TB
PT: Preventive treatment
TB: Tuberculosis
WHO: The World Health Organization.
